# *"I think this is the only challenge… the stigma”* Stakeholder perceptions about barriers to Antenatal care (ANC) and Prevention of mother-to-child transmission (PMTCT) uptake in Kano state, Nigeria

**DOI:** 10.1371/journal.pone.0232028

**Published:** 2020-04-27

**Authors:** Osasuyi Dirisu, George Eluwa, Eseoghene Adams, Kwasi Torpey, Oladapo Shittu, Sylvia Adebajo

**Affiliations:** 1 HIV and AIDS Program, Population Council, Abuja, Nigeria; 2 LEEDx Africa, Abuja, Nigeria; 3 College of Health Sciences, University of Ghana, Accra, Ghana; 4 Department of Obstetrics & Gynaecology, Ahmadu Bello University Teaching Hospital, Zaria, Kaduna, Nigeria; Moi University School of Medicine, KENYA

## Abstract

**Background:**

Despite the progress made so far in reducing mother-to-child transmission (MTCT), Nigeria still contributes significantly to the global burden of new pediatric HIV infections. The elimination target for MTCT has not been reached and the decline in new infections among all Global Plan countries from 2009 to 2015 was lowest in Nigeria. This qualitative study explores the barriers to uptake of prevention of mother-to-child transmission (PMTCT) intervention in Kano, the second most populous state in Nigeria.

**Methods:**

Key informant interviews (KIIs) were conducted among twelve stakeholders who were purposively selected based on their knowledge and involvement in PMTCT program activities in the state. The KII guide explored the status and challenges of PMTCT uptake in Kano state. Qualitative data analysis was managed using NVIVO 11 software and themes were analyzed using thematic analysis.

**Results:**

We found that the key barriers to uptake of PMTCT identified by stakeholders cut across the domains of the socio-ecological model. These include—fear of stigma associated with being seen accessing HIV related services, low male partner involvement, socio-cultural beliefs about the dangers of hospital-based delivery, poor attitude of health workers, distance/cost to facilities, issues with availability of HIV test kits and poor organization of health services.

**Conclusion:**

The implementation of effective PMTCT programs would require innovative strategies that leverage improvement of Antenatal care (ANC) uptake as an entry point for PMTCT. In addition, sustaining engagement in care requires creating a supportive stigma-free environment in the community as well as spousal support to ensure women can navigate the socio-cultural barriers that limit access to health services.

## Introduction

Globally, an estimated 37.9million [32.7million–44.0 million] people were living with HIV in 2018 [1]. The prevalence of HIV in Nigeria (2018 estimate) is 1.4% and it is estimated that 1.9 million people are HIV-infected with women accounting for about 58% of the burden [2,3]. Nigeria contributes significantly to the global burden of pregnant women living with HIV and about one-quarter of mother-to-child transmission (MTCT) globally occurs in Nigeria [4,5]. Nigeria is considered a high burden country for new HIV infections among children and is one of the 22 priority countries which account for 90% of pregnant women living with HIV [6]. These countries were included in the Global Plan to eliminate new HIV infections among children by 2015 [7,8]. Despite the progress made in reducing the burden of pediatric HIV infection in the country, the highest number of new infections among children globally (41,000 [28,000–57,000]) in 2015 occurred in Nigeria [9]. The reduction in new infections among children was only 21% between 2009 to 2015 in Nigeria, the lowest observed among all Global plan countries [10].

Nigeria has one of the highest maternal mortality ratios in the world and contributes the largest proportion of pregnancy-related (postpartum, still births and neonatal) deaths globally [11] and accounted for almost a quarter (23%) of global maternal deaths in 2017 [12]. Nigeria recorded 67,000 maternal deaths in 2017, which was the highest for any country; three other countries (all in Africa) had over 10 000 maternal deaths namely, the Democratic Republic of the Congo (16 000), Ethiopia (14 000) and the United Republic of Tanzania (11 000) [12]. Sixty-one countries were estimated to have had just 10 or fewer maternal deaths in that year. Although this report estimated a Maternal Mortality Ratio (MMR) of 917 per 100,000 livebirths for Nigeria in 2017, the 2018 Nigeria Demographic and Health Survey (NDHS) reported 556 per 100,000 live births [12,13]. Health systems challenges in Nigeria such as poor attitude of health personnel, lack of skilled birth attendants and preference for home delivery conducted by self, family members or traditional birth attendants [TBAs], have been identified as barriers to uptake of Antenatal care (ANC) [14].

Prevention of mother-to-child transmission (PMTCT) interventions can reduce transmission rates from about 45% to less than 5% during the perinatal and postnatal period [15]. Maternal, neonatal and child health (MNCH) services typically provide a platform for delivery of PMTCT; Antiretrovirals (ARV) adherence is linked with ANC attendance [16]. ANC services serve as the first point of contact for pregnant women to engage with the health system and access other services such as HIV testing and PMTCT if diagnosed as HIV-positive [16,17]. This implies that access to health care for pregnant women must be prioritized because an effective ANC program can be used to deliver and improve uptake of PMTCT. Home-based ANC and delivery puts women at greater risk of pregnancy-related complications, vertical transmission of HIV and poor access to ARV [10]. The 2016 Nigeria Multiple Indicator Cluster Survey (MICS) showed that over two-thirds of women registered for ANC in health facilities but over half of these deliveries were home-based in rural areas. Findings from the 2018 NDHS showed that 66% of births to urban mothers were assisted by a skilled provider and 61% were delivered in health facilities, as compared with 29% and 26%, respectively, of births to rural women. Access to skilled ANC was also much lower in rural than urban areas.

PMTCT coverage was barely 30% in Nigeria in 2013 indicating high unmet need for MTCT services [10,18,19]. Barriers to uptake of PMTCT include distance to health facilities, poor attitude of healthcare workers, stigma, poverty and unavailability of services [6,20]. The purpose of this qualitative study was to explore the barriers to uptake of PMTCT intervention in Kano state.

## Methodology

### Study design

This qualitative study was conducted as part of a project that explored the use of an integrated community-based model for provision of MNCH services. The goal was to increase access and retention of pregnant women in the antenatal-postnatal continuum-of-care to facilitate the delivery of PMTCT interventions. Key informant interviews (KIIs) were conducted among stakeholders to explore the status and challenges of PMTCT interventions in Kano State.

### Study setting

The study was conducted in May 2013 in Kano State, the most populous northern state in Nigeria. Kano state is situated in the North-West Zone of Nigeria and is administratively divided into 44 Local Government Areas (LGAs). The state has a population of 9.4million people; females constitute 48.3% of the total population and about half of the female population are women of child-bearing age (15–49 years) [21,22]. The Kano State Ministry of Health (KNSMOH) superintends healthcare in entire State, including service provision in Primary and Secondary Health Facilities (PHCs and SHCs). Whereas the services of SHCs are coordinated by a Kano State Hospitals Management Board (KNHMB), those of PHCs are overseen by the Kano State Primary Healthcare Development Agency (KNSPHCDA). In each LGA, healthcare is supervised by the respective Local Government Department of Primary Healthcare. The project from which this study is derived was implemented in Kura LGA, a rural community in Kano State.

### Study participants and data collection procedure

KIIs were conducted with 12 stakeholders drawn from KNSMOH (2), KNSPHCDA (1), Kura Local Government Department of Primary Healthcare (1), PMTCT program implementing partners (3), service providers (3) and community leaders in Kura LGA (2). Three trained qualitative interviewers with experience in recruitment, conducting interviews, research ethics and stakeholder engagement conducted the KIIs. The interviewers had no prior relationship with the stakeholders. Stakeholders were purposively selected based on their knowledge and involvement with PMTCT program activities in the state. The stakeholders were identified during engagement meetings with heads of agencies organized by the Project Lead to discuss the project objectives. These participants were subsequently selected from the list of stakeholders based on their involvement with PMTCT programs. All participants accepted to participate and the interviews took place in stakeholders’ offices with only the interviewer and stakeholder present. A semi-structured KII guide was developed to stimulate discussions about access to HIV testing as well as barriers and facilitators of uptake of PMTCT. The interviews lasted between 45–60 minutes.

### Data analysis

The interviews were recorded digitally, transcribed verbatim, and transferred to NVivo 11 software to organize the data for analysis. Two researchers read all the transcripts to familiarize themselves with the data and develop the codebook. The researchers reviewed the data and contributed to the development of a thematic framework of codes through consensus. The analytical strategy used was thematic analysis to explore patterns and themes within the data. Some codes were determined as priori codes and others emerged during the coding process. The process of identifying themes highlights contextual situations that underpin perceptions and experiences expressed in the data. The themes were organized using the social ecological model (SEM) to explore perceived barriers of PMTCT including individual level factors such as knowledge and attitudes to PMTCT; interpersonal factors such as HIV status disclosure to spouse and family members; community factors such as stigma and social norms and institutional factors such as organization of health services and manpower challenges. Stakeholders participated in a dissemination meeting where the findings and recommendations were discussed.

### Ethical considerations

The study protocol received ethical approvals from FHI 360’s Review Board, U.S.A and the National Health Research Ethics Committee (NHREC), Nigeria. The procedures involving human participants complied with FHI 360’s Review Board and the NHREC ethical standards for the conduct of research. Written informed consent was obtained from stakeholders by trained interviewers prior to commencement of the interviews.

## Results

**Fig 1:**
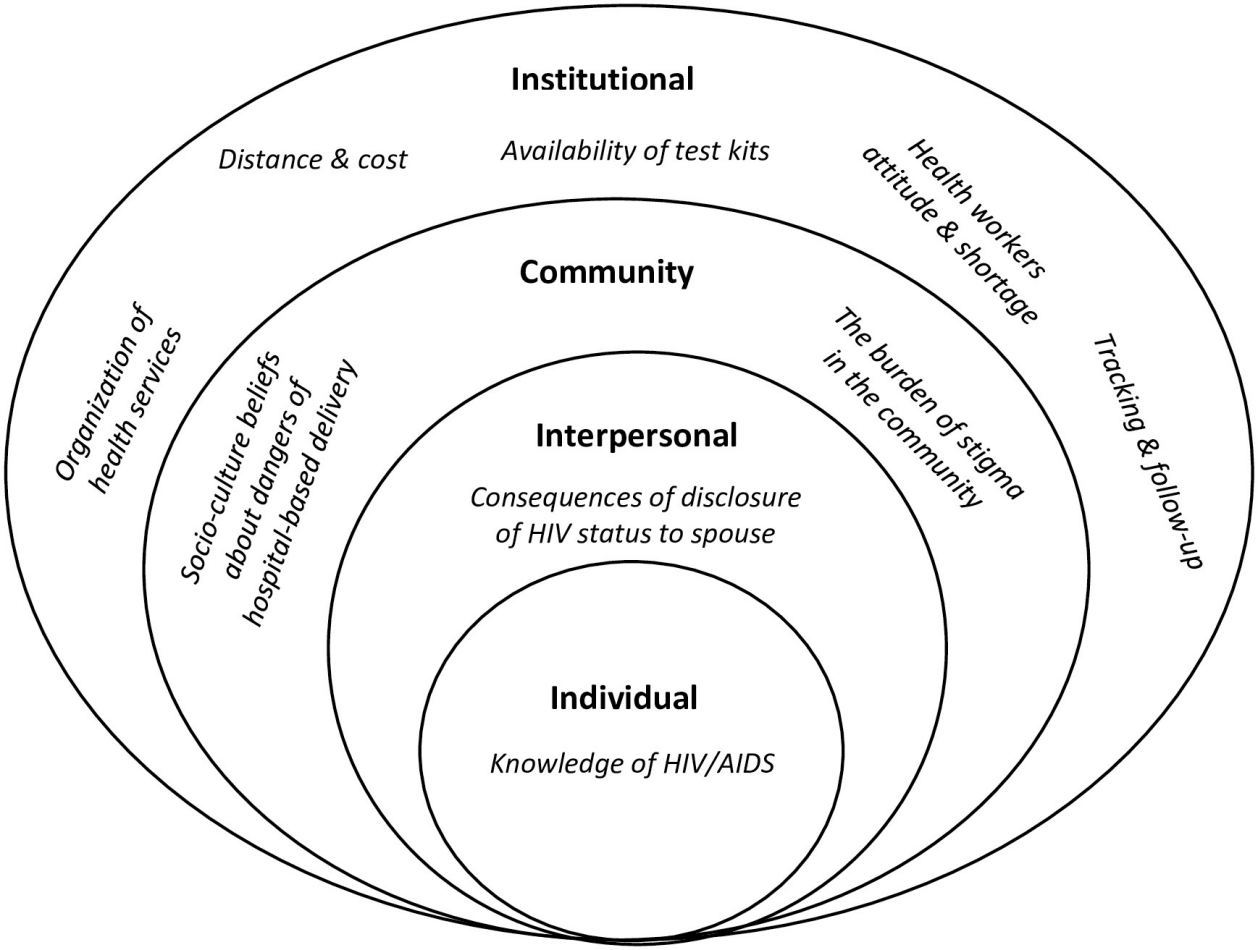
Social ecological model of barriers to PMTCT in Kano state, Nigeria.

The results are presented using the SEM domains: individual, interpersonal, community and institutional. Direct quotes from the interviews are used to illustrate the results at each level as shown in Fig 1. Except for the community leaders, other stakeholders have performed PMTCT-related responsibilities in their organizations such as sensitization of ANC registered women on PMTCT services, supervision of PMTCT services in health facilities, provision of PMTCT services counselling, treatment and coordination of support groups.

### Individual—Level factors

#### Knowledge of HIV/AIDS

There was a consensus among stakeholders that awareness about HIV/AIDS was high in communities across the state. However, awareness did not translate to accurate knowledge about transmission, prevention, care and treatment.

“*The level of awareness is high*. *To me*, *there is hardly any community that you will enter and will talk of HIV/AIDS*, *even in local terminology they all know*. *They must have heard at least once or twice about it…I can’t believe that there is anybody now in the community that you say (ask about) HIV/AIDS or ‘kanjamau’ [Hausa slang for HIV] …that will not know it*. *But awareness of ways of getting infected*, *transmitting it…getting (i*.*e*. *knowing) your status …preventing it*, *treatment and care*, *that one I can say is… low”*.

***KII_State Health Authority***

### Interpersonal factors

#### Consequences of disclosure of HIV status to spouse

Most participants felt women were afraid of knowing their status because of the fear of their husbands getting to know their HIV status. The reluctance to disclose their status was driven by the fear of being sent away from their homes or divorced by their husbands. This fear is aggravated by the fact that most women in these communities are economically reliant on their husbands as they are not engaged in any vocation.

“*They are afraid if… the test turns out to be positive, how are they going to tell their spouse? So that’s the issue because I remember when we started this program earlier in this hospital hmmm… it was a big challenge because many people, many spouses have been divorcing their wives so we have to strategize*.”

***KII_State Health Authority***

### Community level factors

#### The burden of stigma in the community

Stigma was cited as the main reason why women did not want to be involved in PMTCT programmes. The use of designated places as HIV service centres, ARV pick-up or PMTCT clinics was considered stigmatizing because people who accessed services from those clinics were at the risk of being seen by community members visiting the clinics for other services and consequently labelled as HIV positive patients.

“*Presently, I have a client that is positive and she delivered in this facility and there is a Patient Card Issuer here in the hospital she knows; she said she will not come to this hospital just because of that one (person)… and secondly she’s afraid her husband will divorce her if he eventually becomes aware that she’s positive and that is what happened. He divorced her later*.”

***KII_PMTCT Service Provider_Health Facility***

“*The idea of fear, stigma, can prevent them and the setting where the testing is done, we are hoping that not only PMTCT, HIV services should be part of routine, there should be no designation: this is HIV clinic, this is PMTCT clinic*.”

***KII_State Health Authority***

In addition to not wanting to be seen visiting the PMTCT clinic, women were reluctant to join HIV support groups to conceal their HIV status. Keeping away from HIV-related services was their only way of avoiding speculations about the reasons why they visited the clinics and sought care.

“*We have up to 1,800 patients that we have registered here and out of them, I don’t even think up to 200 of them joined support group, and out of 200 I don’t think up to 40 or 50 that is the maximum number that I always see there… they don’t want other people to know that they are in this thing so…*”

***KII_PMTCT Service Provider_ Hospital***

“*She can go to any of the hospitals, but sometimes these hospitals in the remote areas, clients cannot access simple counseling and testing… if she comes out…it will be a problem, ‘ah this woman, what made her go to that place’ ‘maybe she is positive’, I think this is the only challenge… the stigma*.”

***KII_PMTCT Service Provider_Hospital***

#### Socio-cultural beliefs about the dangers of hospital-based delivery

Poor uptake of ANC and delivery attended by skilled birth attendants was reported by some participants as linked to superstitious beliefs about the implications of hospital delivery practices. For instance, socio-cultural beliefs about the consequences of adopting the lithotomy position during delivery impairs women’s decision accept skilled birth delivery. Orthodox obstetric and midwifery practice requires pregnant women to be placed in the dorsal position (lying flat on their back and facing upward, with their lower limbs spread apart and flexed at both hips and knees), to allow the safe emergence of the newborn from the birth canal under the watchful eyes of the care-giver. This practice alienates pregnant women and their relatives from using orthodox maternity services on account of beliefs and fears that pregnant women could die during childbirth by “facing upward to stare into the face of the “angel of death”. These beliefs result in fears among pregnant women that they could die during childbirth in the health facilities resulting in them opting for home-based delivery instead.

“*You know in the hospital when a woman comes to deliver, she has to be in the lithotomy position where she lies on her back while culturally… with their own superstitious belief, they believe (that when) she lies on her back she’s facing the sky and the angel of death is there and if her eyes meet with his, obviously she’s giving up the spirit (she will die). So that’s why they prefer to stay at home to deliver because she would deliver using squatting position*.”

***KII PMTCT _Implementing Partner***

Women who delivered their babies in the hospital were stigmatized as weak; hospital based delivery was considered a sign of weakness because ‘strong women’ gave birth at home.

“*Culture plays a vital role because up till now you see there are people that don’t believe that a woman comes to the hospital and deliver. They feel maybe it is because the woman is weak… and doesn’t want to withstand the shame at home that’s why she comes to the hospital to deliver*.

***KII PMTCT _Implementing Partner***

In addition, religious beliefs played an important role in the decision-making process for ANC uptake. For religious reasons, it was considered unacceptable for ANC and delivery to be supervised by a male health worker even though only male health workers were available in some settings. A community leader reported that some men preferred to accept the fact that their wives died as martyrs than to allow a male health worker to supervise their delivery. In addition, women who delivered at home were supported by prayers. As ANC is a key entry point for PMTCT services, factors that limit access of women to ANC services invariably limit PMTCT.

“*This our culture of privacy…I was surprised to hear that in our primary healthcare centers, there are centers that ANC are being run by males, are you aware? Antenatal care, palpation …by a male, you understand? Delivery the same thing. Sometimes it is the person in charge of the facility, who is a male…and this our notion in the community, ‘wallahi’ (by Allah) for a man to allow his wife… for her delivery to be taken by a male …he had rather leave her at home to die, they will say ‘tayishahada’ (she died as a martyr)*.”

***KII_Community Leader***

*At home-based delivery, the person writing (medicinal Quranic script) is doing it, the person supplicating (prayers) is there in one corner doing it*.

***KII_State Health Authority***

### Institutional level factors

#### Organization of health services

Segregation of services in clinics made it obvious when HIV-related services were available. According to a stakeholder, it was difficult for women to access HIV services anonymously because they received HIV care and ARVs from designated places known for HIV care.

“*The issue of the distance between the ANC and the pharmacy…They have to cross the road to the main pharmacy, and even in the pharmacy, their window is separate from other patients… yeah, it’s a separate place that they collect their ARVs, like say ‘this is the place where you buy drugs, you just go to that place and get your ARVs’… actually people might start thinking*.”

***KII_PMTCT Service Provider_Hospital***

Poor coordination of roles among healthcare providers was reported by some participants as responsible for making the process of obtaining ARV prescriptions and picking up the medication cumbersome. In addition, gaps in training of doctors to manage PMTCT programs as well poor supervision of PMTCT service providers were identified as barriers to effective service delivery.

“*Another challenge is the bureaucracy of prescription in most of our facilities. Ideally, it is the doctor, that is supposed to prescribe ART… for treatment or for prophylaxis. You know most of our ANC clinics are not being run by doctors, so once these nurses test them and they get them positive, they have to run look for a doctor… to prescribe, and some of the doctors, they say they are not trained on PMTCT, not all the ‘gynecologist’ doctors that prescribe*.”

***KII_State Health Authority***

“*Why can’t you monitor? There’s no structure, you understand. In the past (it was) supported by a donor project which when it ran out it (the program) stopped, the EJISS, they called it ‘enhanced joint integrated supportive supervision’ bringing together all program areas to monitor different aspects…*”

***KII_PMTCT Service Provider_Hospital***

#### Distance and cost

The distance patients had to travel to health facilities was considered a major barrier to access to PMTCT services. Patients who enrolled for PMTCT services found it difficult to meet up with appointments because of travel distance. In addition, many women could not afford the cost of transportation to the facilities and the cost of ANC services. Sometimes, women had to leave their homes a day ahead to avoid arriving late for their appointment.

“*There are instances that I housed some women in my house… for the day because they came from a far distance, for them to go and come back the next day or another day, to come as early 7am to access PMTC… Sometimes they have to sleep in my house, the next day I will bring you in the morning you do your test and you go back …*”

***KII_State Health Authority***

“*You discover that even those that are found to be HIV positive… even transport money to come to the facility is a big challenge*.”

***KII PMTCT _Implementing Partner***

“*That is the problem and most of the women don’t have the money to pay for ANC services, so they prefer to stay at home till they have*.”

***KII_Community Leader***

#### Availability of test kits

The reliance on donor agencies for the supply of HIV testing meant that the kits were only available in donor supported facilities. This made access to PMTCT services challenging for women who attended ANC in facilities where test kits were not being provided. Some centers that provided PMTCT services but had shortage of test kits charged a fee for the kits. Unfortunately, the cost of the test kits and transportation to facilities were additional barriers to testing for women who could not afford the cost.

“*Up till now, kits supply to the state is mainly by donor agencies SACA [the State Agency for the Control of AIDS] is supposed to supply all the kits. So if a pregnant woman wants to get tested in Kano, if she’s lucky, she’s attending antenatal care where PMTCT services are provided, she gets tested free of charge otherwise in our local government where [there is] no PMTCT services in majority of our ANC clinics, there’s nowhere, unless she comes to the township, go to a general hospital, she will get tested*.”

***KII_State Health Authority***

“*But now we are having the problem of test kit among others because if I say that every pregnant woman must be tested the numbers of test kits these implementing partners are giving to facilities, is not enough. That is why we even advice our next provider that if the free kits finish, let them buy the kit, continue charging some amount (fee) so that other people will not be left out*.”

***KII_State Health Authority***

#### Health workers’ attitudes and shortage of staff

There was consensus among participants that the negative attitude of health workers discouraged women from accessing ANC or PMTCT services. In comparison, during home-based delivery, women received personalized care from family members and care givers making it more attractive than hospital-based delivery.

It was, however, acknowledged that due to shortage of health workers, facility coverage was poor and service providers could not provide the focused care needed by women during ANC and delivery.

“*This our attitude of the healthcare workers…When women come to deliver at the hospital, they are not given the right care, attention that they deserve. At home-based delivery… you will see those that love you, you understand? They (family) all gather around you, everybody on his feet trying to support… But in the hospital, you are left alone, until after hours, if you are lucky somebody will come around. So that attitude, maybe the healthcare workers they have so much work or they are not enough to take care of the number of patients, to be by the side of every woman delivering*.”

***KII_State Health Authority***

“*Well we don’t have them enough [workers]. You go to a facility you see only two nurses or two midwives managing a whole building. Maternity which comprises of antenatal [clinic], antenatal ward, postnatal ward… only two people running that. So is not easy so the staffs are already overwhelmed so the only way is if we can get more hands*.”

***KII_State Health Authority***

HIV testing and counselling was also considered time consuming because limited staff in health facilities providing a wide range of services resulted in long waiting hours. There was no motivation for women to wait in the facility for HIV services.

“*Counseling and testing is time demanding (consuming), you may spend up to one hour in a particular one…a woman will come early in the morning, she doesn’t leave until late in the evening due to high volume of load in a facility*.”

***KII PMTCT _Implementing Partner***

“*The psychological preparedness may not be optimal for them… the long waiting time …actually affects their cooperation to stay behind and collect ARVs*.*”*

***KII PMTCT _Implementing Partner***

#### Tracking and follow up

The issue of follow up was considered critical in the uptake of PMTCT services and treatment adherence. Patients sometimes provided wrong contact information to conceal their HIV status if they perceived that their spouses could find out about their status in the process.

“*Tracking is a major challenge…is the most critical challenge we have faced in PMTCT. Sometimes, when you have counseled the client, the client will give you a wrong address. When you go, you will not see her? If you go she will say she doesn’t have phone number. Do you have your husband phone number? She will also refuse to give that. She will give a wrong phone number you will call. They will say this is a wrong phone number*.”

***KII_PMTCT Service Provider_Health Facility***

## Discussion

This qualitative study described stakeholders’ perception about barriers to PMTCT uptake in Kano state, Nigeria and the implications for ANC and PMTCT programming. ‥We found that the key barriers to the uptake of PMTCT identified by stakeholders cut across the domains of the socio-ecological model. These include fear of stigma associated with being seen accessing HIV related services, low male partner involvement, socio-cultural beliefs about the danger of hospital-based delivery, poor attitude of health workers, distance to facilities, issues with availability of test kits and poor organization of health services. These findings are consistent with barriers to access to PMTCT services that have been reported in other studies [16,23–26].

Similar to findings in other studies, awareness about HIV was not sufficient to facilitate uptake and adherence [26,27]. General awareness about HIV does not translate to accurate knowledge of HIV testing, transmission, prevention, treatment and care. Anticipated stigma and discrimination at community level affects the willingness of women to access HIV related services at the facilities.

The current service delivery structure lacks confidentiality and stigma is a major barrier to uptake of PMTCT programs [20]. Creating integrated service points as opposed to designated areas for ANC and PMTCT services or ARV pickup may be a valuable strategy to address this issue. The value of PMTCT service integration with other MNCH services in developing countries such as Nigeria has been recommended to facilitate convenience, efficient resource utilization, reduce stigma and improve uptake and retention in care [28,29]. Integration of HIV and ANC services may increase enrollment in HIV care for pregnant HIV+ women [30]. Community sensitization on HIV related issues could be an important step in reducing stigmatization at community level and encouraging disclosure to family members who would support the PMTCT process and facilitate retention [19].

Concerns related to disclosing HIV status to spouses of pregnant HIV+ women include fear of divorce, stigma and ostracism as seen in this study. Unfortunately, adherence to PMTCT services without spousal support is difficult because women need the co-operation and permission of their husbands to attend clinics. Similar findings about disclosure of HIV status to husbands leading to divorce have been reported in other studies [20,31]. Failure to heed husbands’ instructions about ANC and PMTCT was reported as a factor that could lead to divorce in a study in Malawi [32]. In addition, spouses may not want to be tested for HIV or support the treatment process; this non-involvement of spouses has been documented [32,33]. Partner Involvement prior to ANC and PMTCT may be beneficial in ensuring that women receive the support they need for enrollment in care [19]. Women who are ostracized and experience abandonment with no emotional or financial support are not likely to continue treatment [34,35].

Socio cultural beliefs about the delivery process and privacy concerns about supervision of delivery by male health workers should be addressed through education and adaptive strategies that allay the fears of women and their male partners. Women may be more inclined to opt for home-based delivery despite the risks involved if they perceive that facility-based services contradict social norms [20,33]. Decision making about the ANC and delivery process is largely outside the control of the woman and they cannot independently decide to be enrolled in a PMTCT program [20].

Institutional level factors such as poor coordination of health services, shortage of test kits and shortage of staff were found to be critical barriers to PMTCT access. The availability of services may be skewed in favor of donor-supported facilities implying that women must travel far distances to access services if they are not available in their communities. Poorly coordinated services that result in long waiting times, shortage of staff and supplies, congestion and lack of privacy at health facilities have been documented as challenges for PMTCT access [36–38]. Task shifting, logistics planning and community-based mobile PMTCT service delivery have been recommended as strategies to address structural challenges [37]. Successful PMTCT service delivery is hinged on addressing institutional barriers to access [33,39,40].

Strategies to improve uptake of PMTCT services must address issues relating to retention in care and adherence. As participants suggested, potential concerns with tracking and follow-up are hinged on the inability of caregivers to obtain the correct address and phone numbers of women counselled for PMTCT. Underlying the inclination of women to provide the wrong contact details and decline disclosure of their spouses’ contact details is the fear of inadvertent disclosure of their HIV status to their spouses in the process of contact tracing. Increasing male partner involvement in the process is imperative and this has been documented as a critical factor for increasing uptake of PMTCT [24].

## Study limitations

The study was based on stakeholders’ perceptions about uptake of ANC and PMTCT by women. These perceptions may not fully reflect the views of women. A small purposive sample of stakeholders was used and may lack broader generalizability. Findings from this research, however, provided critical insights on the barriers for PMTCT and important guidance on strategies to address them.

## Conclusion

Most of the barriers to uptake of PMTCT were hinged on issues around stigma, sociocultural beliefs and poor organization of health services. The implementation of effective PMTCT programs require innovative strategies that leverage improvement of ANC uptake as an entry point for PMTCT. In addition, ensuring that engagement in care is sustained requires creating a supportive stigma-free environment in the community and spousal support to ensure women can navigate the socio-cultural barriers that limit access to health services. Future research that deepens the understanding of social norms for healthcare decision making as well as male involvement in women’s health will be useful to guide health promotion practice.
